# Natural or artificial: An example of topographic spatial distribution analysis of mescaline in cactus plants by matrix-assisted laser desorption/ionization mass spectrometry imaging

**DOI:** 10.3389/fpls.2023.1066595

**Published:** 2023-02-10

**Authors:** Jiaman Lin, Shuo Yang, Jiaojiao Ji, Ping Xiang, Lina Wu, Hang Chen

**Affiliations:** Department of Forensic Toxicology, Shanghai Key Laboratory of Forensic Medicine, Academy of Forensic Science, Shanghai, China

**Keywords:** mescaline, *Lophophora williamsii*, MALDI-MSI, spatial distribution, *Trichocereus pachanoi*

## Abstract

**Introduction:**

Differentiating whether plant products are natural or artificial is of great importance in many practical fields, including forensic science, food safety, cosmetics, and fast-moving consumer goods. Information about the topographic distribution of compounds is an important criterion for answering this question. However, of equal importance is the likelihood that topographic spatial distribution information may provide important and valuable information for molecular mechanism study.

**Methods:**

In this study, we took mescaline, a substance with hallucinogenic properties in cacti of the species *Trichocereus pachanoi* and *Lophophora williamsii*, as an example to characterize the spatial distribution of mescaline in plants and flowers by liquid chromatograph-mass spectrometry–matrix-assisted laser desorption/ionization mass spectrometry imaging at the macroscopic, tissue structure, and even cellular levels.

**Results:**

According to our results, the distribution of mescaline in natural plant was concentrated on the active meristems, epidermal tissues, and protruding parts of *Trichocereus pachanoi* and *Lophophora williamsii*, while artificially spiked *Lophophora diffusa* products showed no such difference in their topographic spatial distribution.

**Discussion:**

This difference in distribution pattern allowed us to distinguish between flowers that could synthesize mescaline on their own and those that had been artificially spiked with mescaline. The interesting topographic spatial distribution results, such as the overlap of the mescaline distribution map and micrographs of the vascular bundles, were consistent with the synthesis and transport theory of mescaline, indicating the potential for applying matrix-assisted laser desorption/ionization mass spectrometry imaging in botanical research.

## 1 Introduction

The family Cactaceae includes a wide variety of succulent plants that are native to the American continent but have been introduced to almost all other continents, mainly for ornamental purposes (Fontenele et al., 2021). Several cactus species, including *Trichocereus pachanoi* (San Pedro cactus) and *Lophophora williamsii* (peyote cactus), contain a hallucinogenic alkaloid called mescaline(β-3,4,5-trimethoxyphenethylamine) (Ghansah et al., 1993; Halpern, 2004). However, other species in the same genera as these plants do not contain mescaline—an example is *Lophophora diffusa*.

Mescaline is a secondary metabolite thought to play a role in preventing browsing by herbivores during plant growth; however, the relationship between the presence of mescaline and particular cactus species has not been well studied. In humans, mescaline acts as a serotonin receptor agonist with affinity for 5-HT_1A_ and 5-HT_2A/B/C_ serotonin receptors, with a main action on 5-HT_2C_ receptors(Monte et al., 1997; Harms et al., 2003; Pálenícek et al., 2008). It is a phenethylamine analogue, and mescaline intoxication produces psychosis-like symptoms, including changes in mood and sensory perception, disturbances of thought and the visual sphere, impaired tactile perception, hypersensitivity to noises, alterations in motor and physical perception, changes in spatial and temporal perception, synesthesias, and various hallucinations (Cassels and Sáez-Briones, 2018; Nichols and Walter, 2021). Some mescaline users can experience suspicions that can develop into paranoid delusions (Carstairs and Cantrell, 2010; Uthaug et al., 2022). Mescaline products come in relatively small doses (parts per million level) compared to traditional drugs such as meth (~70 percent). This makes mescaline easier to be transported without being seized by traditional anti-drug agencies.

Purified mescaline crystals are classified as a Class A drug in the UK and as a controlled Class I psychotropic substance in China^1^. *L. williamsii*, in its dried form, is controlled in Canada, the Netherlands, and Germany. In the United States (Lee et al., 2020) and Korea^2^, both mescaline itself and the *L. williamsii* plant are listed as controlled. Unfortunately, these differences in the legal control of natural plants and mescaline crystals in different countries and regions provide a “grey area” for some international drug gangs. They can buy inexpensive, common horticultural varieties of cactus, cultivate them, and then soak the flowers of the horticultural cactus varieties in a solution of dissolved mescaline crystals. This stains the petals with mescaline, but the flowers with very low mescaline content (parts per million levels) may evade customs DNA and anti-drug tests, allowing the illegal transport of the drug. The low cost of the artificial products and the high return made selling these “natural products” can bring huge illegal profits.

In terms of anti-drug work, tracking down and combatting the source of mescaline are more important than knowing whether the seized items contain drugs. Knowledge of whether the drugs are naturally occurring or have been artificially added to plants can help establish the direction of an investigation. Studying the topographic spatial distribution of compounds in a plant sample may therefore be advantageous. More importantly, understanding the topographic spatial distribution of natural compounds in plants is useful in botanical research. For example, a high-throughput quantitative method has been used to analyze phytohormone levels in *Sorghum bicolor* leaf and root tissues (Dong et al., 2012), allowing the analysis of plant growth and the proposal of valuable growth-induction schemes. Studies on the spatial distribution of the polyphenol content in leaf, stem, and root extracts of *Humulus scandens* (Choi et al., 2018) can also improve the understanding of optimal harvesting sites and further culture schemes when seeking the raw materials for antioxidants. Most of the current methods used for detecting mescaline are typical analytical methods, such as gas chromatography-mass spectrometry (GC-MS) (Henry et al., 2003; Pálenícek et al., 2008; Gambelunghe et al., 2013) and liquid chromatography-mass spectrometry (LC-MS) (Kikura-Hanajiri et al., 2005; Sergi et al., 2013; Boumba et al., 2017). Researchers have used qualitative and quantitative analyses of the target compounds in different sampling areas to deduce the spatial distribution of the target compounds in the whole plant.In recent years, mass spectrometry imaging (MSI) has become increasingly used in the field of botany (Boughton and Thinagaran, 2018; Shimma and Sagawa, 2019; Hou et al., 2022; Ikuta et al., 2022). MSI provides a much finer spatial resolution compared to traditional analysis methods, such as LC-MS. In addition, it allows the *in situ* visualization of a large number of biomolecules in the sample while retaining the original sample shape (Enomoto and Miyamoto, 2021), and indicates the spatial distribution of relevant metabolites during plant growth and development (Liao et al., 2019). For example, MALDI-MSI revealed that the accumulation of toxic quinolizidine alkaloids (QAs) in seeds of the high protein content narrow-leafed lupin (NLL, *Lupinus angustifolius)* was mainly transported by other tissues, and the results open the possibility of using transport engineering to generate herbivore-resistant bitter NLL varieties that produce QA-free seeds (Otterbach et al., 2019). Study on the distribution of highly toxic ergot alkaloids, ergocristine and ergometrine, produced by Ecc93 and Gal 310 variants of fungus *Claviceps purpurea* after infection of rye renders MALDI-MSI a powerful tool for investigating biosynthetic pathways and for obtaining a deeper understanding of the parasite-host interaction(Dopstadt et al., 2017). When used in conjunction with optical microscopy, MSI can combine morphological features with chemical graphs to provide a targetless, label-free, and versatile method for molecular imaging (Nie et al., 2021). The high instrument sensitivity, high spatial resolution, high accuracy of detection quality, and fast determination speed are significant advantages; consequently, matrix-assisted laser desorption/ionization mass spectrometry imaging (MALDI-MSI) is now showing great potential for the imaging of endogenous molecules in tissue sections in botany (Sturtevant et al., 2016; Qin et al., 2018; Susniak et al., 2020; Sun et al., 2022).

As a soft ionization technique, MALDI involving laser irradiation of a sample section sprayed with a matrix. The matrix absorbs the laser energy and transmits it to the sample molecules, a charge transfer and sublimation occur, and the material enters the mass spectrometer. The mass spectral signal is obtained by a detector, the data obtained are converted into pixel points by imaging software, and an image of the spatial distribution of the target compound on the surface of the tissue is constructed (Yalcin and de la Monte, 2015).

In the present study, LC-MS and MALDI were used to analyze the natural spatial distribution of mescaline in *T. pachanoi* and *L. williamsii*. The objective was to analyze the natural distribution pattern of mescaline in whole cactus plants, focusing on petal structure and microstructure. The topographic spatial distribution of mescaline in artificial plant products was also compared with the natural distribution by an artificial preparation experiment. The overall goals were to understand the differences in the distribution of mescaline in natural plants and artificial products and to analyze the causes of natural distribution according to current theories.

## 2 Experimental

### 2.1 Materials

#### 2.1.1 Chemicals

Mescaline and 25B-NBOMe-D_3_ (internal standard) were purchased from Cerilliant (Texas, USA). HPLC-grade methanol (MeOH) and acetonitrile (ACN) were purchased from Sigma-Aldrich (St. Louis, MO, USA). MALDI grade trans-2-[3-(4-tert-butylphenyl)-2-methyl-2-propenylidene) malononitrile (DCTB, >98.0%) was purchased from TCI (Shanghai) Development Co. Ltd (Shanghai, China). HPLC-grade dichloromethane (DCM) was purchased from CNW, ANPEL Laboratory Technologies Inc. (Shanghai, China). Indium tin oxide (ITO)-coated glass slides (dimensions 75 × 25 × 1 mm, sheet resistivity ≤ 5 Ω) and double-sided carbon conductive tape (dimensions 12 mm ×20 m, resistivity < 5 Ω/mm^2^), used for MALDI-MSI, were purchased from South China Science & Technology Co. Ltd. (Shenzhen, China) and SPI supplies (West Chester, USA), respectively. Ultra-purified water was prepared using a Milli-Q water purification system (Millipore Corp., Burlington, MA, USA).

A standard stock solution of mescaline (10 μg/mL) was prepared in MeOH and diluted to the desired concentrations before use. A working internal standard solution (10 ng/mL) was prepared by dilution in methanol. A matrix solution of DCTB was prepared by dissolving 15 mg of DCTB powder in 50 µL DCM and then adding 1.45 mL ACN.

#### 2.1.2 Cactus samples

**Figure 1:**
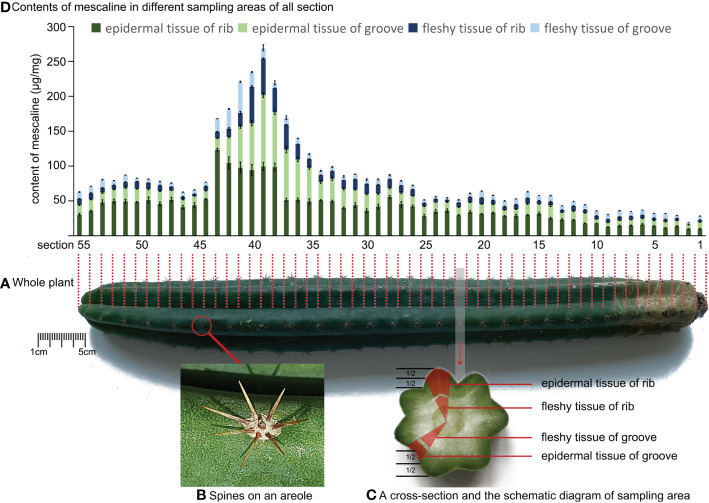
**(A)** Schematic diagram of the position of the longitudinal incision of *Trichocereus pachanoi*. **(B)** Spines on an areole of *Trichocereus pachanoi*. **(C)** A cross-section and the schematic diagram of sampling area of *Trichocereus pachanoi*. **(D)** Mescaline content in different sampling areas of all sections of *Trichocereus pachanoi*.

A *T. pachanoi* sample was supplied by customs officials, who had discovered five whole *T. pachanoi* plants hidden in a package trying to clear customs. According to the principle of random sampling, one of the five plants was selected and sent to our laboratory by the anti-drug law enforcement department for further analysis (**Figure 1A**). The *T. pachanoi* sample was an erect columnar cactus about 45 cm long overall, with a green crown about 40 cm long at the top and a brown subterraneous stem about 5 cm long at the bottom. It had 7 ribs, each with an even distribution of 27 areoles from top to bottom. The areoles were hairless, with needle-like spines (**Figure 1B**).

**Figure 2:**
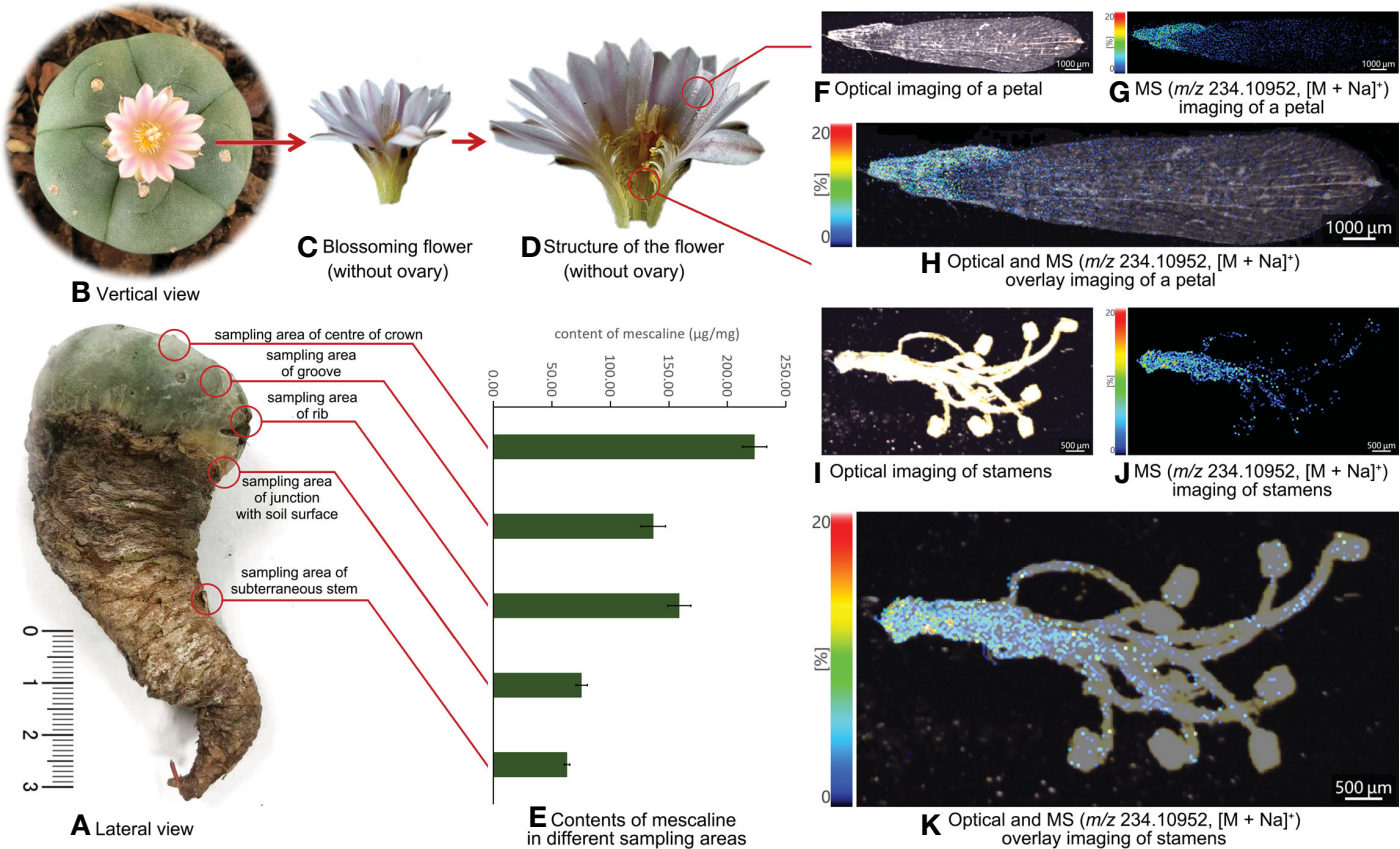
**(A)** Lateral view of *Lophophora williamsii* and the different sampling areas. **(B)** Vertical view of *Lophophora williamsii*. **(C)** The blossoming flower of *Lophophora williamsii* (without ovary). **(D)** Internal structure of the flower of *Lophophora williamsii* (without ovary). **(E)** Mescaline content in different sampling areas of *Lophophora williamsii*. **(F)** Optical imaging of the petal of *Lophophora williamsii.* **(G)** MS imaging of the petal with a resolution of 30 µm (*m/z* 234.10952, [M + Na]^+^). **(H)** Optical and MS overlay imaging of the petal. **(I)** Optical imaging of stamens of *Lophophora williamsii.* **(J)** MS imaging of stamens with a resolution of 30 µm (*m/z* 234.10952, [M + Na]^+^). **(K)** Optical and MS overlay imaging of stamens.

A *L. williamsii* sample was also supplied by customs officials, who had found 12 whole *L. williamsii* plants hidden in a package trying to clear customs. According to the principle of random sampling, one of the 12 plants was selected and sent to our laboratory by the anti-drug law enforcement department for further analysis (**Figure 2A**). The alive *L. williamsii* sample was an oblate, spineless cactus with a gray-green color. The crown of the *L. williamsii* sample had 5 ribs, averaging 4.72 ± 0.064 cm wide and 2.9 cm high, with 3 areoles on each rib. The areoles were spineless and hairy. The center of the crown was a specialized flower-bearing area of the cactus, densely woolly or hairy, with short, spiny internodes. The *L. williamsii* bloomed several times after a period of cultivation (**Figure 2B**). Its flowers consisted of two peripheral organs (sepals and petals) occurring in two outer whorls, and two inner reproductive organs (stamens and pistils) (**Figures 2C, D**). These organs were arranged in a concentric pattern in the floral meristem, which was fused downward into a connate perianth that protects the ovary inside. The ovary contained multiple ovules. The flower petals were pink to white and averaged 1.79 ± 0.04898 cm in length.

**Figure 3:**
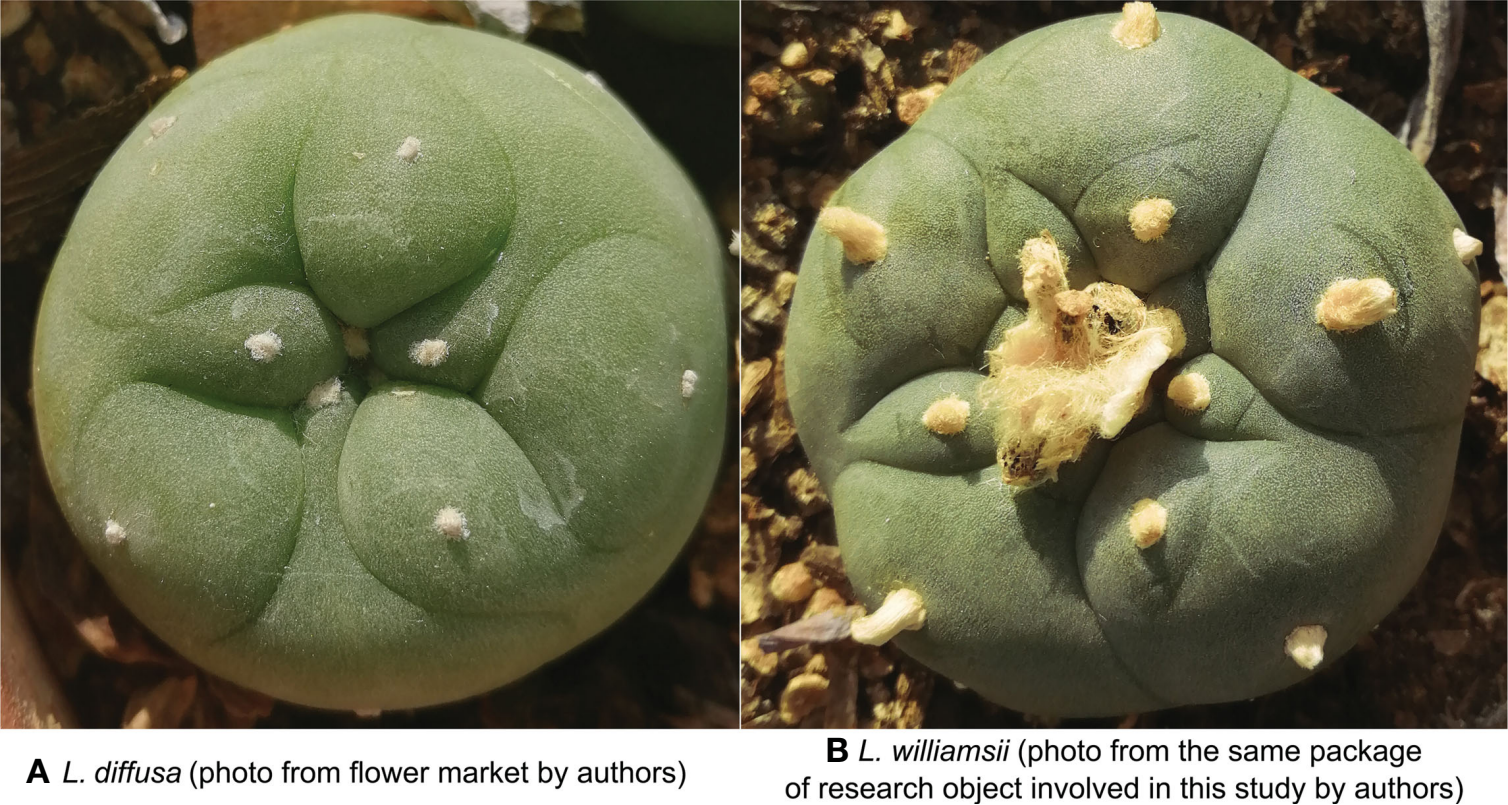
**(A)** Vertical view of *Lophophora diffusa* (photo from flower market by authors). **(B)** Vertical view of *Lophophora williamsii* (photo from the same package of research object involved in this study by authors).

The *L. diffusa* sample was purchased from a local flower market. Judging by appearance alone, non-experts would find it hard to believe that *L. diffusa* and *L. williamsii* are different species (**Figure 3**). Even their different flower colors could be attributed to different horticultural varieties by the general public. The *L. diffusa* in this study also bloomed several times after a period of cultivation. The *L. diffusa* was yellow-green, had 13 ribs with 4 areoles on each rib, and the crown averaged about 7.37 ± 0.099 cm wide and 5.3 cm high. Its petals were white to yellow and averaged 1.46 ± 0.072 cm in length.

Some of the *L. diffusa* petals were plucked to prepare “artificial” petals by spiking. Naturally dried *L. diffusa* petals were placed on a slide and spiked with a standard methanol solution of mescaline (1 μg/mL) using a pipette and evenly spotting the solution on the petal surface. Once the petals were dry, the mescaline in part of the spiked petals was quantified by LC-MS. The spiking procedure was repeated until the “artificial” petals had a mescaline content of about 30 ng/mg. This spiking content is to mimic natural *L. williamsii* petals. For the analysis, six dried *L. williamsii* petals were selected and quantified by LC-MS. Their mescaline contents ranged from 26.25 ng/mg to 34.19 ng/mg; therefore, we chose 30 ng/mg as the spiking mescaline content.

All three cactus samples underwent DNA tests to ensure that they belonged to the appropriate species.

### 2.2 Sample preparation

#### 2.2.1 Sample preparation for LC-MS

The *T. pachanoi* sample was cut into 55 equal sections, from the top of the crown to the subterranean stem, as shown by the red dashed line in **Figure 1A**. Each interval section contained at least one areole. The epidermal and fleshy tissues of the rib and groove were sampled from each section (**Figure 1C**). All spine samples (n=27) were also collected along one rib. To exclude the influence of the tissue water content on the quantitative results, the sampled tissues were dehydrated as follows: The epidermal tissues, fleshy tissues, and spine samples were put into a -80°C refrigerator for 12 h for pre-freezing and then lyophilized (LyoQuest, Telstar, Spain) at -59°C with a vacuum of 0.040 mBar for 48 h. The resulting structurally fluffy, internally dry, spongy, soft, and elastic lyophilized samples were then used for subsequent tests. After lyophilizing, all tissue samples were extracted as described previously (Yang et al., 2022). Lyophilized tissue (20 mg) was weighed (Cubis II Micro Balance, Sartorius, Germany) into a 2 mL tube with ceramic beads, followed by the addition of 40 µL IS (10 ng/mL) and 660 µL methanol. All samples were pulverized by a JXFSPRP-CLN freeze-grinder (Shanghai Jing Xin Industrial Development Co., Ltd., China) at below −35°C. The mixture was ultrasonicated for 10 min and then centrifuged at 13,500 rpm (11,610xg) for 5 min. After centrifugation, 300 µL of supernatant was filtered through a 0.22 µm filter membrane (Sinopharm Chemical Reagent Co., Ltd., China), and a volume of 5 µL of the filtrate was injected into LC-MS.

The *L. williamsii* sample was sampled from five different positions: groove, rib, center of the crown, the junction with the soil surface, and the subterranean stem (**Figure 2A**). Half of each sampled section was separated into epidermal and fleshy tissues. The five intact tissue and separated tissue sections were dried as described for *T. pachanoi*.

An additional fleshy tissue sample was collected from the crown of *L. williamsii*. This section was used to extract chloroplasts by grinding in 10 mL sodium chloride solution (0.35 mol/L), then filtering the homogenate through two layers of gauze. The filtrate was centrifuged at 1000 rpm (860xg) for 2 min, and the pellet was discarded. The supernatant was centrifuged again at 3000 rpm (2,580xg) for 5 min to pellet the chloroplasts. The discarded pellet from the first centrifugation and the supernatant from the second centrifugation were combined and used as a control sample. The chloroplast preparation and the control sample were dried under nitrogen and then extracted as described for the *T. pachanoi* samples. The same extraction method was also used for the spiked *L. diffusa* petals.

#### 2.2.2 Sample preparation for MALDI-MSI

Fresh *L. williamsii* flower samples were collected on the second day of flowering and divided into four parts (sepals, petals, stamens, and pistils) with tweezers under an optical microscope (Carl Zeiss, Göttingen, Germany). The flat sample surface required by MALDI analysis was obtained by treating each part as follows: The pistils were first cut in half. The sepals, petals, stamens, and pistil halves were then flattened, sandwiched between two layers of air-laid paper (Kimberly-Clark Professional, USA), and pressed in a book to make a “bookmark” (Safa et al., 2022). The bookmarked sepals, petals, stamens, and pistil halves were then pasted onto the surface of ITO-coated conductive glass slides with double-sided carbon conductive tape. An 800 μL volume of the DCTB matrix solution was added to the cavity of an artist’s airbrush (MR Linear Compressor L7/PS270 Airbrush, Tokyo, Japan) and used to spray the matrix uniformly onto the surface of the samples on the slides. The distance between the tip of the airbrush and the tissue surface was about 8 cm. After spraying, the samples were left at room temperature for 5 min to allow solvent vaporization. The same sample preparation was used for the natural *L. diffusa* petals and spiked *L. diffusa* petals.

### 2.3 Instruments

#### 2.3.1 Imaging based on stereomicroscopy

The cell structure of the plant tissues were examined under a stereo microscope (Zeiss Axio Imager Z2, Göttingen, Germany), using the imaging software AxioVision SE64 (Carl Zeiss, Göttingen, Germany) to control the instrument and image acquisition. K-Viewer Digital Slide Reading Software (Ningbo Konfoong Bioinformation Tech Co., Ltd., China) was used to analyze the images.

#### 2.3.2 LC-MS

The LC–MS system consisted of an Acquity™ ultra-performance LC system (Waters Corporation, USA) equipped with an AB Sciex6500 Q-trapTM triple quadrupole mass spectrometer (AB Sciex, Foster City, USA). Analyst 1.6.3 software (AB Sciex, Foster City, USA) was used to control the system and collect the data, and MultiQuant 3.0.2 software (AB Sciex, Foster City, USA) was used to analyze the data. The mescaline content in different samples was determined using a previously published LC-MS method, the detection limit of method was 0.003 μg/mg and the intra batch precision was 8.8% (Yang et al., 2022). SPSS 25.0 (IBM, USA) was used for statistical calculations.

#### 2.3.3 MALDI-MSI

All MALDI-MSI data were acquired on an iMScope QT instrument (Shimadzu, Kyoto, Japan), which has an integrated optical microscope (magnification ×5, ×10, and ×40), an atmospheric pressure MALDI source, and an ion trap/quadruple time-of-flight (IT-TOF) analyzer. The scanning area was determined using an optical microscope, and the sample slices were irradiated with a diode-pumped 355 nm Nd : YAG laser using the following parameters: laser shots, 200; repetition rate, 2000 Hz; laser diameter, 0 (5 μm); laser intensity, 45; detector voltage, 2.4 kV; pitch (spatial resolution), 30 μm. For mescaline analysis, the instrument was operated in positive mode, and spectra were acquired in the range of *m/z* 50 to 400. The laser was calibrated with ink, and an accurate mass was ensured by calibrating the MALDI-MSI instrument with the DHB matrix before each experiment. All acquired data were analyzed using IMAGEREVEAL MS software (Shimadzu, Kyoto, Japan). The same software was used for visualization and relative quantitative analysis. The tolerance (the window of mass range) for MALDI-MSI was set at 5 ppm to exclude interference by ions adjacent to the target ion. SPSS 25.0 (IBM, USA) was used for statistical calculations.

## 3 Results

### 3.1 Topographic spatial distribution of mescaline in whole plants

The topographic spatial distribution of mescaline in *T. pachanoi* is shown in **Figure 1D**. The mescaline content in *T. pachanoi* ranged from 0.14 ± 0.01 μg/mg to 102.6 ± 3.49 μg/mg. The mescaline content was significantly lower (independent sample T test, P<0.05) in the subterranean stem (0.14 ± 0.0 μg/mg to 16.05 ± 1.35 μg/mg) than in the crown (2.78 ± 0.22 μg/mg to 102.6 ± 3.49 μg/mg). In the whole plant, the peak mescaline content was detected in sections 38 to 43 (mean content=102.88 ± 10.55 μg/mg) and was almost 2 times higher than in the adjacent sections in the rib area (mescaline content in section 37 = 51.17 ± 2.51 μg/mg, content in section 44 = 52.74 ± 1.11 μg/mg).

We used the double-sample difference method to calculate the content differences in the same tissues in the rib and groove of the same cross-section. We first subtracted the content of mescaline in the rib from the content in the groove. The difference was then divided by the mean content of mescaline in the rib and groove. We then took the absolute value of the quotient, which ranged from 13.0% to 183% (> intra batch precision of the method [8.8%]; Yang et al., 2022). The same method was used to calculate the differences in mescaline content between epidermal and fleshy tissues in the rib and groove. The differences in mescaline content between epidermal and fleshy tissues were also greater than the intra-batch precision of the method, with a maximum difference of 191%. The mescaline content was higher in epidermal tissue than in fleshy tissue (independent sample T test, P<0.05). The mescaline contents in the spines of *T. pachanoi* were all below the detection limit of 0.003 μg/mg.

A similar topographic spatial distribution was also observed in *L. williamsii* (**Figure 2E**): the mean mescaline content in the groove, rib, center of crown, junction with the soil surface, and subterranean stem was 136.68 ± 10.47 μg/mg, 158.94 ± 9.81 μg/mg, 223.24 ± 10.33 μg/mg, 75.36 ± 5.03μg/mg, and 62.95 ± 2.39 μg/mg, respectively. The mescaline content was higher in epidermal tissue than in fleshy tissue (independent sample T test, P<0.05).

### 3.2 Topographic spatial distribution of mescaline in flowers

**Figure 4:**
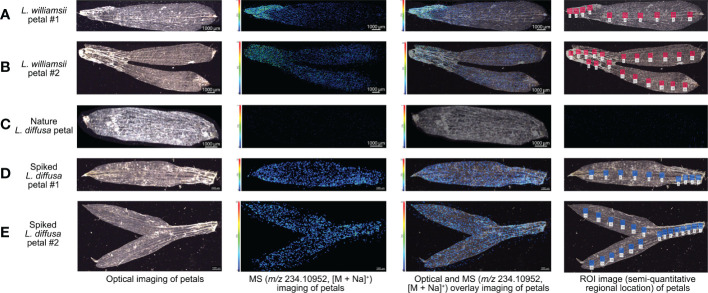
Optical imagings, MS imagings with a resolution of 30 µm (*m/z* 234.10952, [M + Na]^+^), optical and MS overlay imagings and ROI semi-quantitative regional location of mescaline analysed by MALDI-MSI in positive ion mode in different petal samples. **(A)** Petal sample 1 of *Lophophora williamsii*. **(B)** Petals sample 2 of *Lophophora williamsii*. **(C)** Petal sample of *Lophophora diffusa*. **(D)** Spiked petal sample 1 of *Lophophora diffusa*. **(E)** Spiked petals sample 2 of *Lophophora diffusa*.

The MALDI-MSI analysis of petals, sepals, stamens, and pistils of *L. williamsii* flowers revealed that mescaline (*m/z* 234.10952, [M + Na]^+^) was concentrated in the stalk region of the petals (close to the receptacle) (**Figures 2F-H**). IMAGEREVEAL MS software was used to conduct a regions of interest (ROI) semi-quantitative analysis, and the value of each ROI showed the relative intensity of the average spectrum (pixel by pixel) of mescaline produced by MALDI-MSI analysis in a given region (**Figures 4A, B**). A total of 10 ROIs (each ROI contained 250 pixels) of the same size were selected for each petal to calculate the relative intensity, with 4 ROIs on the stalk region. For double petals, 20 ROIs were selected, with 8 ROIs on the stalk region. The mean deviation of the ROIs for the *L. williamsii* petals was 98.8, with a range of 265.92. The mean ROI of the stalk region was 285.73, and that of the tip region was 19.81. Unlike the mescaline content of the flower petals, mescaline (*m/z* 234.10952, [M + Na]^+^) was concentrated in the tip region of the sepals (away from the receptacle). In the pistil and stamens (**Figures 2I-K**), the mescaline content was higher in the style than in the stigma.

The same method was used to obtain MALDI-MSI data for spiked *L. diffusa* petals (**Figures 4D, E**): the mean deviation of the ROIs of the spiked petals was 26.0, with a range of 67.40. No mescaline signal was detected in unspiked *L. diffusa* petals (**Figure 4C**).

### 3.3 Topographic spatial distribution of mescaline in microstructure

**Figure 5:**
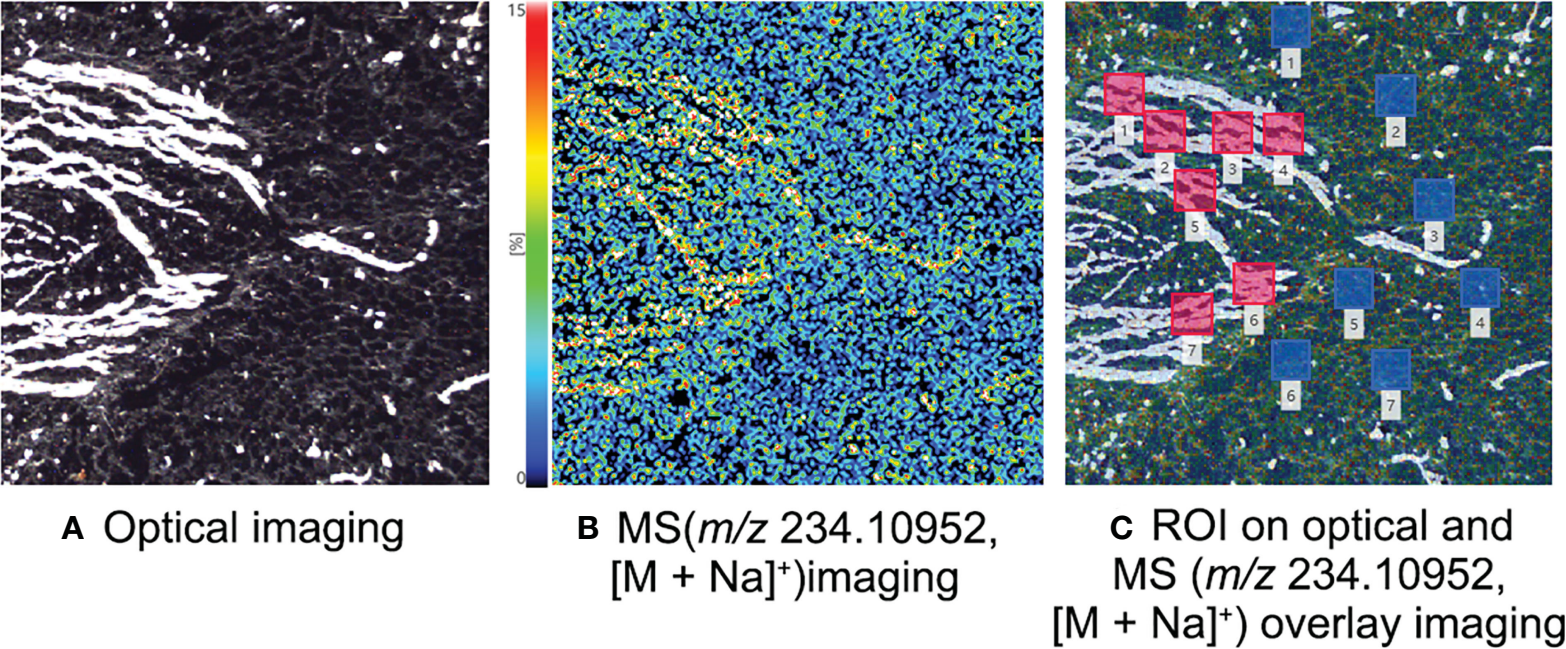
MALDI-MSI analysis of mescaline in the subterranean stem slice of *Lophophora williamsii*. **(A)** Optical imaging. **(B)** MS imaging with a resolution of 30 µm (*m/z* 234.10952, [M + Na]^+^). **(C)** ROI semi-quantitative regional location on optical and MS overlay imaging.

The subterranean stem slices of *L. williamsii* were analyzed by selecting a specific slice with concentrated vascular bundles. The slice was analyzed by MALDI-MSI with a spatial resolution of 30 μm (**Figure 5**). A total of 7 ROIs (each ROI contains 250 pixels) of the same size were selected for vascular and nonvascular regions to calculate the relative intensity. The mean relative intensity of the ROIs of the vascular bundle was 1444.51 ± 236.63, while mean intensity of the ROIs of the nonvascular area was 632.21 ± 68.18. The relative intensity in the ROI was significantly higher in the vascular bundle region than in the nonvascular region (independent sample T test, P<0.05).

Chloroplasts isolated from the crown of *L. williamsii* by gradient centrifugation had an average mescaline content of 7.40 ± 1.99 μg/mg (n=3), while the average content of the rest of the extract was 120.22 ± 7.10 μg/mg (n=4).

## 4 Discussion

The fine sampling, complicated sample preparation, and LC-MS analysis performed here allowed us to obtain approximate topographic spatial distributions of mescaline in whole plants of *T. pachanoi* and *L. williamsii*. In general, mescaline was not evenly distributed throughout either plant, and was less concentrated in the subterranean stem. The results of the two-sample difference analysis led us to believe that mescaline is more abundant in the ribs than in the grooves. Further analysis of the epidermal and fleshy tissues indicated a potential enrichment of mescaline in epidermal tissues. Other studies also indicated that mescaline is more abundant in the epidermal tissue than in the fleshy tissue (Reti and Castrillón, 1951; Longo and Musah, 2020), in agreement with our MALDI-MSI results. The mescaline content was also reportedly higher in the crown than in stems or roots (Klein et al., 2015), being consistent with the present study. These results support the following theory put forward previously (Fürstenberg-Hägg et al., 2013) that plants minimize the costs of adaptation to damage by building defenses against their enemies and increasing resistance to foraging. The hallucinogenic properties of mescaline cause a transient loss of behavioral ability and place herbivores at higher risk of attack by predators. Therefore, to serve as a defensive compound, mescaline is more concentrated in vulnerable areas, such as epidermal tissues and ribs. Our results are consistent with this theory.

Our results also confirm the value of studying the topographic spatial distributions of specific target compounds in botany, as this can provide a better understanding of various molecular mechanism problems. As shown here, this spatial distribution result provides a linear answer to the mechanical question of where mescaline molecules are concentrated. However, our LC-MS results also show the weakness of traditional research methods. First, the sampling must be accurate. We could not have obtained our results without the availability of fresh whole plants, but this is often not possible in practice in the laboratory. We also need to use a complicated sample preparation process, including careful dehydration to obtain accurate quantitative results, efficient extraction to ensure the accuracy of data, and efficient development and validation of all methodology.

Our MALDI-MSI analysis of the flowers confirmed that the differences in mean deviations indicated a significant difference between the mescaline distribution in *L. williamsii* petals (98.8) versus spiked *L. diffusa* petals (26.0). This answered one of the original questions posed here: whether natural mescaline is distributed differently from artificially spiked mescaline. We found that mescaline was naturally concentrated in the stalk region of the *L. williamsii* petals, whereas the distribution was homogenous in artificially spiked *L. diffusa* petals. This result would be difficult to ascertain using traditional LC-MS methods. First, the weight of the whole petal was only about 0.5 mg, which is difficult to weigh accurately for quantitative LC-MS analysis. Second, although the microsampling technique (Zhou et al., 2020) we developed previously enabled the small-scale sampling of petals, the flow of sap may have interfered with the accuracy of those analysis results. MALDI can achieve semi-quantification of target compounds on a small scale, while *in situ* analysis can avoid errors caused by sampling. The petal analysis in this study demonstrates that the MALDI-MSI technique provides both these advantages.

**Figure 6:**
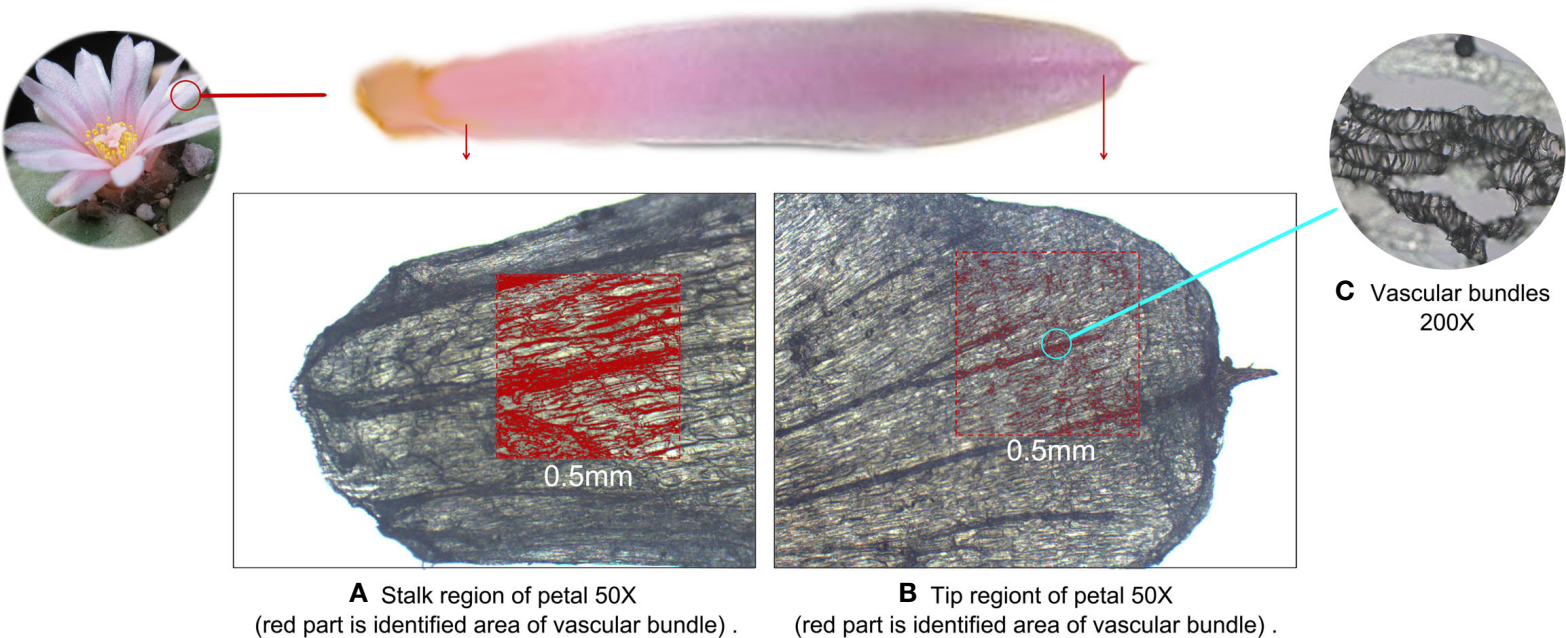
Microscopic optical image of the petal of *Lophophora williamsii*, with the red area defined as vascular bundles. **(A)** Stalk region of the petal. **(B)** Tip region of the petal. **(C)** Vascular bundles (200X).

We further examined the microstructure of the regions in which mescaline was concentrated using the K-Viewer Digital Slide Reading Software to analyze the stalk and tip regions of the petals. The analysis area was 0.5 × 0.5 mm in size. The darker parts were identified as vascular bundles. In the stalk area, 62.3% of the area was identified as vascular bundles (red-marked area in **Figure 6A**), whereas only 9.9% of the area was identified as vascular bundles in the tip region (red-marked area in **Figure 6B**), for an approximately 6.3-fold difference in area. The approximate ROI difference between the two regions was 14.4-fold (285.73/19.81). Higher magnification revealed that the vascular bundles were dominated by ducts (**Figure 6C**). The vascular bundle is essential for plant growth, and its main functions are transport of water, nutrients, and signal molecules and support of the plant body (Yoshioka et al., 2001), suggesting that the topographic spatial distribution of mescaline is related to the vascular bundles.

We examined the relationship between vascular bundles and mescaline topographic spatial distributions by conducting a more detailed MALDI-MSI analysis. We simultaneously imaged mescaline at different areas on the same slide and found a clear overlap of the vascular bundle with the mescaline locus in a smaller area (**Figure 5**). The mescaline content was significantly higher in the vascular bundle area than in the nonvascular area. The average mass spectrum response was 2.3 times higher in the vascular bundle region than in the nonvascular region. Some interesting statistical information was also apparent: the difference in the vascular bundle area was 6.3-fold higher for the stalk than for the tip of the petal. Multiplying this number by the coefficient (2.3) of the multiple of mescaline mass spectrometry responses for the vascular and nonvascular regions gave a result of 14.5, which was fairly close to the ROI multiples of mescaline between the stalk and tip of the petal (14.4).

The combination of the more systematic spatial distribution data of LC-MS and the more refined spatial distribution data of MALDI-MSI allowed us to answer the question of the natural distribution of mescaline in plants. Interestingly, these data may reveal a possible “life cycle” of most mescaline molecules. Mescaline is a well-known secondary metabolite of cacti, and uneven distributions of secondary metabolites are common in plants, as maintaining relatively high levels of secondary metabolites throughout the plant throughout its life cycle is “costly” to the plant, as these require synthesis from simple precursors and then transport to and accumulation in sites of storage or action (Harborne, 1990). The synthesis of mescaline is closely related to amino acid metabolism (Rosenberg and Stohs, 1974), as the generally accepted route of mescaline synthesis is from tyrosine or a hydroxylated phenylalanine *via* decarboxylation and O-methylation (Leete, 1966). The synthetic pathway occurs mainly in newborn cells (Watanabe et al., 2013); therefore, this may explain why mescaline is most concentrated in sections 38 to section 43 of *T. pachanoi* and in the center of the crown of *L. williamsii*. These two areas are also the most concentrated meristem locations in these two plants (Deng and Ashihara, 2015). Although we have attempted a further analysis of the organelles that might produce mescaline, our attempt was not successful using only simple gradient centrifugation. Our results were not completely consistent with the findings of other studies (Ogunbodede et al., 2010; Klein et al., 2015; Dinis-Oliveira et al., 2019).

The mescaline synthesized in the active plant sites then enters the transport process in a similar pattern previously reported (Isah, 2019) and is transported along the vascular bundles to other parts of the plant. In this process, some mechanism likely directs the molecules toward the epidermal tissue, the plant protrusions, and the reproductive organs. In this way, although not involved in plant growth, mescaline plays essential roles in the interactions of plants with their biological environments, mainly against herbivores (Choudhary et al., 2008; Nuringtyas et al., 2012; Cook et al., 2013). There are many studies using MALDI-MSI on the metabolism and molecular mechanisms of plant responses to abiotic and biotic stress and symbiotic relationships. Sarsby et al. reported that in *Arabidopsis thaliana*, glucosinolates accumulated differently in specific cells of reproductive organs and that tissues containing high concentrations of glucosinolates were less likely to be eaten by herbivores (Sarsby et al., 2012). Ritmejerytė et al. revealed a tissue-specific distribution of cyanogenic glycosides (defence metabolites that deter herbivores by releasing hydrogen cyanide upon hydrolysis) in stigma cells and pollen of cyanogenic florets of the genus *Lomatia* and substantial variation in their concentrations within florets suggesting that their allocation is under strong selection (Ritmejerytė et al., 2020). Dreisbach et al. indicated an increased latex flow rate towards the point of damage leading to an accumulation of cardiac glycosides and other defence metabolites in the affected area of the leaves of *Asclepias curassavica *(Dreisbach et al., 2021). Numerous studies have shown that MALDI-MSI is useful in plant science for obtaining molecular information on topographical features at the cellular level, and the fine visualisation of metabolites demonstrates the advantages of MALDI-MSI for the study of plant defence mechanisms.Therefore, taken together, our results support the hypothesis that mescaline is more prevalent in the active meristems, epidermal tissues, and protruding parts of the cactus, which are all areas that are vulnerable to animal browsing. The active meristem is the main place of synthesis of mescaline, while the epidermal tissue and the protruding parts that are vulnerable to animal browsing are the place of storage of mescaline. The plant delivers the mescaline from the place of synthesis to the place of storage in the vessels by a certain transport mechanism. Our results for the topographic spatial distribution analysis by MALDI-MSI indicate many interesting studies to explore in this plant.

## 5 Conclusions

In this study, LC-MS and MALDI were used to study the topographic spatial distribution of naturally occurring mescaline in *T. pachanoi* and *L. williamsii*. We also used *L. diffusa*, a cactus species that does not naturally contain mescaline, as the base material to prepare and compare the topographic spatial distribution of mescaline in artificial plant products. The natural mescaline in *T. pachanoi* and *L. williamsii* was more enriched in the protruding parts, meristems, and epidermal tissues, while the distribution in the artificially manipulated plants showed no such difference. Refined MALDI analysis demonstrated the value of the topographic spatial distribution information of target compounds for mechanistic studies of some plants. The results of MALDI-MSI visualization, which showed an enrichment in the vascular bundle regions, combined with the regional content differences obtained by LC-MS, were consistent with current mescaline production and transport theories. The findings reported here not only answer the original study question, namely, whether plant products from natural or artificial imitation can be distinguished by spatial analysis, but they also demonstrate the potential of topographic spatial analysis based on LC-MS and MALDI techniques for botanical research.

## Data availability statement

The original contributions presented in the study are included in the article/supplementary material. Further inquiries can be directed to the corresponding author.

## Author contributions

Authors JL and SY share first authorship. All authors contributed to the article and approved the submitted version.

## Footnotes

1 National Medical Products Administration (2013) Catalogue of narcotic drugs and psychotropic drugs.(2013). https://www.nmpa.gov.cn/directory/web/nmpa/xxgk/fgwj/gzwj/gzwjyp/20131111120001419.html. [Accessed Sep 7, 2022].

2 Korea Ministry of Government Legislation. Narcotics Control Act. https://www.moleg.go.kr/index.es?sid=a1 [Accessed Sep 7, 2022].
